# Protection of Children in France

**Published:** 1893-06

**Authors:** 


PROTECTION OF CHILDREN IN FRANCE.—The French infant-food bill, which recently has become law, prohibits parents from giving their children under one year of age solid food in any form without the prescription of a physician. The use of bottles with rubber tubes is also forbidden. M. Rouchard, the president of the Society for the Protection of Children, to whose efforts the passing of the above bill is principally due, asserts that out of 250,000 infants annually dying in France 100,000 might be saved by careful nursing.—*Ex.*

